# P-566. Long-term virologic outcomes of oral two-drug vs three-drug antiretroviral regimens in children, adolescents and adults living with HIV

**DOI:** 10.1093/ofid/ofae631.764

**Published:** 2025-01-29

**Authors:** Morgan Byrne, Wei Li A Koay, Justin Unternaher, Lauren O’Connor, Anne Monroe, Rachel V Denyer, Amanda Derryck Castel, Natella Rakhmanina

**Affiliations:** George Washington University, Washington, District of Columbia; Medical University of South Carolina, Charleston, South Carolina; Children's National Hospital, Washington, District of Columbia; George Washington University, Washington, District of Columbia; George Washington University, Washington, District of Columbia; Washington DC VA Medical Center/ George Washington University, Washington, DC; The George Washington University Milken School of Public Health, Washington, DC; Children's National Hospital, Washington, District of Columbia

## Abstract

**Background:**

Despite the availability and efficacy of two-drug regimens (2DR) for HIV treatment, three-drug regimens (3DR) are mostly used by people with HIV (PWH). We evaluated virologic outcomes of initiating or switching to a 2DR vs 3DR among PWH in Washington, DC.

Adjusted Cox Proportional Hazard Regression Model for Virologic Non-Suppression (viral load > 200 copies/mL) among PWH by Two- and Three-Drug Regimens in the DC Cohort, 2019-2023

**Figure:**
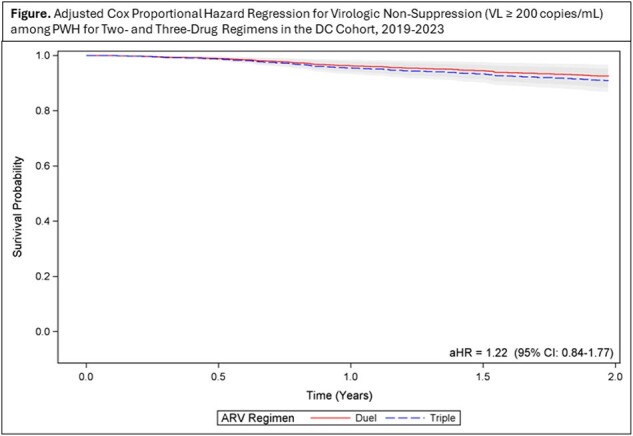

**Methods:**

We included DC Cohort treatment-experienced participants initiating or switching to a 2DR or 3DR on or after July 2019 with ≥2 years of follow-up. Descriptive statistics for demographic and clinical factors at regimen initiation, including HIV viral load (VL) and CD4 count (cells/µL), were stratified by 2DR or 3DR. Multivariable Cox proportional hazard modeling was performed among participants virally suppressed (VS; VL< 200 copies/mL) at regimen initiation to assess the time to non-virologic suppression (non-VS) using adjusted hazard ratios (aHR) and 95% CI adjusting for demographics, HIV risk, and regimen. Among participants who were not VS at the time of regimen initiation, we conducted a sub-analysis of the time to VS.

**Results:**

We analyzed 1782 participants (72.8% 3DR, 28.2% 2DR (161 switched from a 3DR); age 54.3 years (IQR 43.8-61.3), 68.8% male; 78.1% Black) with a median CD4 621 (IQR 409-868) at regimen initiation. Participants on 2DR were older (56.4 vs 53.6 years; p< 0.001) and had higher VS rates (93.8% vs 88.2%; p< 0.001) at regimen initiation and shorter regimen times (3.2 vs 3.8 years; p< 0.001) compared to 3DR. 2DR participants were more likely to have public vs private insurance and receive hospital vs community-based care. The risk of non-VS was higher among those with CD4 < 200 and 200-500 (aHR 1.5(1.0-2.1) and aHR 2.0(1.1-3.6), respectively) compared to those with CD4 >500. Those with public insurance were also more likely to experience non-VS (aHR 1.5; p< 0.05). While regimen type was not a significant predictor of non-VS, there was an elevated risk among those on 3DR (aHR 1.2) compared to 2DR [Figure]. Among those non-VS at regimen initiation (n=146), the median time to VS was similar among PWH on 2DR (14.1 months) and 3DR (13.8 months; p=0.76).

**Conclusion:**

Non-VS PWH have similar time to VS regardless of whether they receive 2DR or 3DR, suggesting similar efficacy of 2DR and 3DR in these populations. Patients with CD4 < 500 had higher risk of non-VS on 2DR, supporting the usefulness of the CD4 criteria factor when selecting 2DR vs 3DR.

**Disclosures:**

**All Authors**: No reported disclosures

